# Correlation of calprotectin serum levels with degrees of endometriosis: A cross-sectional study

**DOI:** 10.18502/ijrm.v19i7.9474

**Published:** 2021-08-16

**Authors:** Raden Muharam, Muhammad Saiful Rizal

**Affiliations:** Department of Obstetrics and Gynecology, Faculty of Medicine, University of Indonesia, Jakarta, Indonesia.

**Keywords:** Calprotectin, Endometriosis, C-reactive protein, Inflammation.

## Abstract

**Background:**

Endometriosis is closely associated with delayed diagnosis due to the lack of a definitive and sensitive noninvasive approach. The use of calprotectin in inflammatory process has been demonstrated in various inflammatory diseases. Calprotectin has a significant correlation with high-sensitivity C-reactive protein (hs-CRP) and could be used as an inflammatory marker. No study thus far has evaluated the correlation between calprotectin and endometriosis.

**Objective:**

To determine the correlation of calprotectin with the degree of endometriosis in order to help clinicians in establishing better early detection and management.

**Materials and Methods:**

In this cross-sectional study, 46 women referred to the Cipto Mangunkusumo, Fatmawati, and Persahabatan Hospitals in Jakarta, Indonesia between July 2017 and April 2018 were enrolled, and their blood serum were taken a day before surgery. Calprotectin serum level was treated using the PhicalⓇ ELISA method. After the diagnosis of endometriosis was confirmed through pathological examination, the final diagnosis of endometriosis could be established. The degree of endometriosis was classified according to the revised American Society for Reproductive Medicine (ASRM) classification.

**Results:**

The prevalence of minimal, mild, moderate, and severe degrees of endometriosis were 15.2, 39.1, 34.8, and 10.9%, respectively. The median serum calprotectin levels for minimal, mild, moderate, and severe endometriosis were 138.98, 121.49, 124.16, and 122.82 mg/mL, respectively. No correlation was observed between calprotectin and the degrees of endometriosis (r = –0.16, p = 0.278).

**Conclusion:**

There is no correlation between calprotectin serum levels and the degrees of endometriosis.

## 1. Introduction

Endometriosis is generally associated with decreased quality of life in patients due to the prolonged and cyclic pain. The symptoms are usually associated with menstruation and chronic pelvic pain (1). Other problems include late diagnosis and the time of endometriosis therapy (around -12 yr) (2). There is no definitive and sensitive noninvasive approach or biomarkers for the early diagnosis of the disease (3). The incidence of endometriosis is difficult to measure, some of these women are often asymptomatic and the imaging modalities have a low sensitivity. Its prevalence among reproductive-age women is between 5 and 10% (4), with an asymptomatic prevalence ranging from 2 to 22% (5). There is no accurate diagnostic method other than looking directly into the pelvis (laparoscopy) and ascertaining it by anatomic pathological examination (6). Inflammation and immune response play an important role in the development of endometriosis; chronic inflammation causes the development of endometrial ectopic tissue growth, influences the immune system, which is related to angiogenesis, lymphangiogenesis, and neurogenesis (7).

Calprotectin is released from neutrophils and monocytes during their activation, and increased systemic levels of the protein have been linked with increased immunological activity and inflammation (8). The use of calprotectin has been demonstrated in various inflammatory diseases, it has the advantage of being stable at room temperature. In addition to serum, calprotectin levels can also be measured in body fluids such as saliva, synovial fluid, or feces (9). Calprotectin has been reported to be correlated with serum high-sensitivity C-reactive protein (hs-CRP) levels, which supports the hypothesis that it could be used as an inflammatory marker (10). To the best of our knowledge, no study has ever examined the relationship between the levels of calprotectin and endometriosis.

The aim of this study was to determine the correlation of serum calprotectin with the degrees of endometriosis according to the Revised 1996 American Society for Reproductive Medicine (rASRM) classification. It is hoped that this study can help clinicians in better early detection and management efforts of endometriosis, so that the prognosis would be better.

## 2. Materials and Methods

This study was conducted at the Cipto Mangunkusumo General Hospital, Fatmawati General Hospital, and Persahabatan Hospital Jakarta between July 2017 and April 2018. The inclusion criteria used were: patients (i) aged 15–46 yr, (ii) who had undergone surgery in one of the aforementioned hospitals, (iii) were diagnosed with endometriosis, and (iv) gave consent to participate in the study. The diagnosis of endometriosis was confirmed after the pathology results showed endometriosis. On the other hand, patients who (i) were diagnosed with diabetes mellitus and/or had random blood glucose > 200 mg/dl, (ii) were diagnosed with hypertension and/or had blood pressure systolic > 140 mmHg or diastolic > 90 mmHg, (iii) were confirmed or suspected of having infection (leucocyte > 10,000 cell/mm${}^{3}$), (iv) were diagnosed with liver disease, and (v) received corticosteroid or immunosuppressant therapy within the last three months from the surgery. The degrees of endometriosis were determined based on the clinician's assessments during the surgery. Accordingly, the categorical variables were divided into minimal (score 1–5), mild (score 6–15), moderate (score 16–40), and severe (score > 40) degrees according to the revised 1996 ASRM classification.

The levels of calprotectin serum were processed by using PhicalⓇ Calprotectin ELISA kit (Immunodiagnostik AG, Bensheim, Germany) with VMaxⓇ Kinetic ELISA Microplate Reader (Molecular Devices, Sunnyvale, CA, USA), and Heraeus LabofugeⓇ 200 centrifuge (Thermo Scientific, Waltham, MA, USA) according to the manufacturer's instructions in the form of numerical variables with an ng/mL measuring scale. Five milliliters of blood serum were taken and processed in the laboratory from 46 patients undergoing a laparoscopic and laparotomy surgery, one day before surgery.

### Ethical considerations

This study was approved by the Ethical Committee of the Faculty of Medicine, University of Indonesia, Jakarta, Indonesia (No: 755/UN2.F1/ETIK/2017). All participants gave a written informed consent prior to the study.

### Statistical analysis

This study used an observational analysis with a cross-sectional design. The sample calculation was done by using formula suitable for the correlative analytics (11). Alpha standard deviation was set at 5%, with a positive two-way research hypothesis (there was a correlation between the calprotectin levels and the degree of endometriosis), and the alpha deviation value was 1.96. Based on the calculation, 46 women diagnosed with endometriosis were needed. Therefore, a consecutive sampling was carried out, and all subjects with endometriosis were included as the research sample until the required number of subjects was met. The degrees of endometriosis had categorical variables divided into minimal (score 1–5), mild (score 6–15), moderate (score 16–40), and severe (score > 40).

The data and research results were recorded in the research sheet, stored on computer, data verification was carried out, and coding was done for data analysis. Calprotectin serum levels in the form of numerical variables (independent variable) and the degree of endometriosis in the form of categorical variables (dependent variable) were reported in the form of univariate and bivariate analysis. This study was analyzed using the SPSS 20.0 computer statistics program software. We used the Spearman's test to measure the correlation between the two variables. The correlation coefficients of 0.10–0.29 showed a weak correlation, 0.30–0.49 showed a moderate correlation, and coefficients > 0.5 showed a strong correlation.

## 3. Results

**Figure 1:**
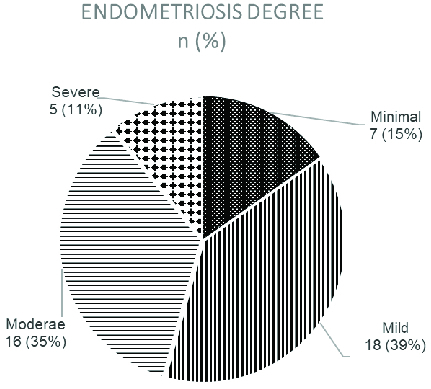
The prevalence of the different degrees of endometriosis.

Blood serum samples were taken from a total of 46 subjects a day before their surgery and the results of pathology anatomy and surgical findings according to endometriosis were traced. Of the 46 subjects, 26 were in the follicular phase of the menstrual cycle, while the other 20 either did not know when their last menstrual cycle was or had forgotten about it, however, none of the subjects were in their menstrual period. Figure 1 illustrates the prevalence of endometriosis.

The correlation test results showed a moderate positive correlation of patients' age and body mass index (BMI) with the degrees of endometriosis (r = 0.37 and r = 0.36), which was statistically significant (p = 0.012, and p = 0.014, respectively) (Table I).

Data were analyzed regarding the correlation between Calprotectin and the endometriosis degrees. Data were in the form of numeric-ordinal variables and the normal distribution of data was tested. Calprotectin data and the endometriosis degrees had normal data distribution (p ≥ 0.05), so a correlation test was carried out by using the Spearman's method. The mean value of calprotectin levels for the minimum degree of endometriosis was 138.98 ng/mL (74.84–434.64), mild degrees was 121.49 ng/mL (11.46–305.16), moderate degrees was 124.16 ng/mL (11.6–368.9), and severe degrees was 122.82 ng/mL (43.92–268.78). To date, no study has compared serum calprotectin in patients with endometriosis. In this study, the correlation test showed that there was no correlation between calprotectin and endometriosis degrees (r = –0.16) and was not statistically significant (p = 0.278) (Table II).

**Table 1:** Relationship between the characteristics of subjects and the different degrees of endometriosis


		**Degrees of endometriosis**				**r**	**p-value**
**Characteristics of subjects**		**Minimal**	**Mild**	**Moderate**	**Severe**		
Age (yr)*		32 ± 8	33 ± 7	37 ± 7	42 ± 4	0.37	0.012${}^{a}$
BMI (kg/m${}^{2}$)**		23.2 (19.6-24.8)	22.6 (15.7-34.7)	24.1 (16.0-34.0)	28.0 (23.9-36.7)	0.36	0.014${}^{a}$
Infertility***							
	**Yes**	6 (85.7)	10 (58.8)	8 (53.3)	2 (40.0)	0.263${}^{b}$
	**No**	1 (14.3)	7 (41.2)	7 (46.7)	3 (60.0)	
Type of infertility***							
	**Primary**	4 (66.7)	7 (70.0)	5 (62.5)	1 (50.0)	0.648${}^{b}$
	**Secondary**	2 (33.3)	3 (30.0)	3 (37.5)	1 (50.0)	
*Data presented as Median ± SD, **Data presented as median (min–max), ***Data presented as n (%). ${}^{a}$Statistical tests using the Spearman's correlation test, ${}^{b}$Statistical tests using the chi-square. r: Correlation coefficient, SD: Standard deviation, BMI: Body mass index							

**Table 2:** Correlation between calprotectin and the different degrees of endometriosis


**Variable**	**Endometriosis **				**r**	**p-value**
	**Minimal**	**Mild**	**Moderate**	**Severe**		
**Calprotectin (ng/mL)**	138.98 (74.84–434.64)	121.49 (11.46–305.16)	124.16 (11.6–368.9)	122.82 (43.92–268.78)	–0.16	0.278
Data presented as median (min–max). Spearman's correlation test						

## 4. Discussion

Chronic inflammation and immune response play a role in the endometriosis development. Calprotectin will increase in conditions of infection and inflammation. In chronic inflammatory conditions, the serum calprotectin level will increase, for example, in rheumatoid arthritis, obesity, and atherosclerosis. At the extracellular, calprotectin has a proinflammatory effect through a dependent toll-like receptor 4 (TLR-4) mechanism. It has been reported that the calprotectin levels were significantly correlated with serum Hs-CRP levels, which shows that calprotectin could be used as an inflammatory marker (r = 0.510, p < 0.001) (10). Another study reported the advantage of calprotectin being stable at room temperature. Calprotectin is a good candidate to monitor activity and the degree of the disease. In addition to serum, calprotectin levels can also be measured in body fluids such as saliva, synovial fluid, or feces. Another advantage of serum calprotectin is that it is stable and easily measured compared to other cytokines, so that calprotectin has the potential to be a good biomarker of inflammatory diseases (9). Calprotectin was also reported as a marker that could be used to monitor the active stage of the rheumatoid arthritis (RA) disease, which is a chronic inflammatory disease of joints. Calprotectin is secreted locally during the inflammatory process and its concentration increases in joint fluid and is also associated with the degree of the disease. It is also said that calprotectin is superior to CRP for predicting synovitis, with a positive correlation between the two (r = 0.556). A moderate-to-strong correlation between serum calprotectin and various classifications of disease severity was obtained (12). Moreover, an association was found between the levels of fecal calprotectin and the severity of atopic dermatitis in children, fecal calprotectin levels were significantly higher in high-grade atopic dermatitis (p = 0.044) and also had a significantly positive correlation with the degree of disease classification with the Scoring Atopic Dermatitis (SCORAD) index system (r = 0.303, p = 0.014) (13).

Calprotectin is a calcium-binding protein which belongs to the S100/calgranulins group. It is a heterodimer of two calcium-binding proteins, that are, S100A9 and S100A8 (14, 15). The concentration of calprotectin is high in neutrophils and is secreted to the extracellular from stimulated neutrophils and monocytes, or it is secreted as a result of cell damage or death. Not only in neutrophils and macrophages, but calprotectin can also be found on the membrane of non-keratinized squamous epithelium. Dissolved calprotectin can be found in blood plasma, urine, body secretions (saliva), intestinal fluids, and feces (16). Calprotectin binds to TLR4 which can explain inflammation and infection (17). S100A8 and S100A9 form complex (calprotectin) that triggers the TLR4-signaling pathway, which in turn increases the proinflammatory cytokines production, including the Interleukin 1β (IL-1β), which then activates monocytes and neutrophils. This activation increases the secretion of S100 proteins (S100A8 and S100A9) so that the calprotectin production also increases and so on (18). There are many advantages of calprotectin testing, but there are no studies that correlate it to endometriosis.

Although endometriosis is a chronic inflammatory disease, there is no correlation between the systemic effect of serum calprotectin and endometriosis. In this study, the results of the serum calprotectin correlation test with the degrees of endometriosis showed no correlation between the two and were not statistically significant. As is well-known, menstruation is an inflammatory process. Liu and colleagues found that various inflammatory cytokines were greatly increased and reached their highest levels during the menstrual period, whereas the lowest levels were found during the follicular phase of the menstrual cycle (19). Moreover, 26 of the 46 subjects (56%) in this study were in the follicular phase of the menstrual cycle, and the remaining 20 were unknown because the subject either did not know when the last period was or had forgotten about their menstrual cycle, but the subjects were certainly not in a menstrual period. The results of calprotectin serum for various degrees of endometriosis (minimal, mild, moderate, and severe) showed the respective medians of 138.98 ng/mL, 121.49 ng/mL, 124.16 ng/mL, and 122.82 ng/mL. In another study using control patients, the median normal serum calprotectin level was 1318 ng/mL (range: 215.8–3770 ng/mL) (11). The results of this study further support that markers in peripheral serum are still controversial and there is no evidence to suggest any marker that can be used for the diagnosis or monitoring of endometriosis (20). Local inflammation will sustain growth and development of endometriosis through endometrial-peritoneal adhesions, invasion, angiogenesis, and proliferation (2). Activation of inflammatory cells in ectopic endometrial tissue induces angiogenesis, lymphangiogenesis, and neurogenesis. There are dysfunctional immune cells found in peritoneal fluids such as macrophages and natural killers, these immune cells fail to eliminate endometrial ectopic cells (7).

## 5. Conclusion

No correlation was found between calprotectin serum levels and the degrees of endometriosis. Further studies need to be done to examine the correlation between fecal and peritoneal fluid calprotectin as a marker of local inflammation with the degrees of endometriosis.

##  Conflict of Interest

The authors declare that they have no conflict of interest to report.
